# Novel Screening Methods for Binary Outcomes with Rare Events

**DOI:** 10.21203/rs.3.rs-10925760/v1

**Published:** 2026-10-09

**Authors:** Mohammad Nahian Ferdous Abrar, Hongmei Zhang, Meredith Ray, Yuanjin Liu, Hasan Arshad, Yu Jiang

**Affiliations:** 1 Division of Epidemiology, Biostatistics, and Environmental Health, School of Public Health, University of Memphis, Memphis, TN, USA; 2 Maryville University, Saint Louis, MO, USA; 3 David Hide Asthma and Allergy Research Centre, Isle of Wight, UK; 4 Human Development and Health, Faculty of Medicine, University of Southampton, Southampton, UK

**Keywords:** rare event outcomes, ttScreening, resampling, sensitivity, specificity

## Abstract

**Background:**

Epigenome-wide association studies often involve high-dimensional DNA methylation data. Efficient methods are needed to screen CpG sites associated with binary outcomes, particularly when the event rate is below 5%. Existing screening methods, such as *ttScreening*, are effective for filtering out unimportant variables, such as CpG sites, in epigenome-wide studies when event rates are moderate. However, *ttScreening* and many other screening approaches face challenges in handling rare events. Therefore, we proposed two novel methods, *Rare-Screening* and *Firth-ttScreening*, designed specifically for binary outcomes with rare events.

**Results:**

We evaluated the performance of the proposed approaches and benchmark methods, including FDR-based and Bonferroni approaches for multiple testing correction, through extensive simulations across varying sample sizes and numbers of independent variables.

**Conclusions:**

The *Rare-Screening* and *Firth-ttScreening* methods outperformed FDR and Bonferroni methods with higher sensitivities. The proposed functions have been incorporated into the *ttScreening* R package, which is available through the Comprehensive R Archive Network (CRAN).

## 1 Background

Epigenome-wide association studies (EWAS) typically evaluate hundreds of thousands of cytosine–phosphate–guanine (CpG) sites in a single analysis, leading to an ultra-high-dimensional screening problem. When the outcome of interest is binary and rare, such as a disease or developmental event occurring in only a small proportion of participants, this problem becomes more challenging. The number of CpG sites is very large, whereas the number of event cases is often too small for stable single-CpG logistic regression across all sites. As a result, conventional association testing can produce unstable regression coefficients, inflated standard errors, low power, and convergence problems, particularly when the event rate is below 5%.

In rare-event EWAS, an effective screening method should reduce the CpG search space while retaining a high proportion of truly associated markers. The CpG sites selected by such a procedure should therefore be interpreted as candidate markers for downstream analysis, such as biological pathway analysis, prediction modeling, independent validation, or formal multiple-testing correction, rather than as definitive discoveries with guaranteed false discovery rate (FDR) control. This distinction is important because screening and inference have different goals: screening prioritizes retaining potentially relevant variables, whereas formal inference prioritizes calibrated error control.

Several multiplicity adjustment methods have been developed for high-dimensional testing. A classical approach is the Bonferroni correction, which controls the family-wise error rate by setting the per-test significance threshold to be *α* divided by the total number of hypotheses. However, this correction is widely recognized as overly conservative, with substantial loss of power and an increased risk of missing true associations, especially when effect sizes are modest [1, 2]. False discovery rate (FDR)-based methods aim to control the expected proportion of false discoveries among the rejected hypotheses. The Benjamini–Hochberg (BH) procedure, for example, orders p-values from smallest to largest and rejects those below an adaptive threshold that depends on their rank [2]. Storey’s direct approach to FDR further introduced adaptive estimation of the false discovery rate and q-values, providing a less conservative alternative in many large-scale testing settings [3]. When hypotheses have known or suspected structure, such as genomic clustering, annotation-defined groups, or covariates, structure-adaptive FDR procedures may improve power by upweighting regions more likely to contain signals and downweighting predominantly null regions. Compared with Bonferroni-type family-wise error rate control, FDR procedures are generally less conservative. However, in high-dimensional settings with many tests but relatively weak signals, the rejection threshold will be very small when the number of tests is large, so only the strongest effects are selected [4]. This limitation becomes more pronounced when the outcome is rare, and the effective number of cases is small.

Variable screening provides a complementary strategy for ultra-high-dimensional data. Rather than making final inferential claims for all CpG sites, screening aims to remove clearly uninformative variables and retain a smaller set of candidates for downstream analysis. Sure independence screening (SIS) has been widely used to filter irrelevant independent variables and reduce ultra-high-dimensional feature space to a manageable size [5]. SIS and related approaches are commonly based on marginal association measures, and their value lies in retaining important variables with high probability before applying more computationally intensive or confirmatory methods. However, because marginal screening ranks variables one at a time, it may retain irrelevant variables that show strong marginal but not necessarily joint associations with the outcome, particularly in noisy or small-sample settings [6].

A complementary strategy for screening in high-dimensional settings is to use sample splitting or resampling to assess the stability of selected variables. This idea is closely related to stability selection, which combines subsampling with high-dimensional selection algorithms and retains variables that are selected consistently across repeated samples [7]. Dobbin and Simon [8] examined such sample-splitting schemes for high-dimensional classifiers and showed that allocating approximately two-thirds of the sample to training and one-third to testing performs well on datasets with sample sizes of at least 100, providing a good compromise between power and the reliability of performance estimates. Building on this training–testing principle, Ray et al. proposed the *ttScreening* procedure for high-dimensional DNA methylation studies [9, 10]. In *ttScreening*, DNA methylation level at each CpG site is modeled as the response, while phenotype and relevant covariates are included as predictors. Candidate CpG sites are selected in the training set and later evaluated in the testing set, and this procedure is repeated across multiple iterations. CpG sites that repeatedly pass the screening criterion are retained. This framework makes *ttScreening* computationally efficient and useful as a preprocessing step. However, when the outcome is binary, and events are rare (< 5%), this setup becomes problematic: the case group is very small, so the sample size for estimating the mean difference in methylation between cases and controls is limited, leading to unstable regression coefficients, inflated standard errors, and low power to detect truly associated CpGs. As a result, *ttScreening* may fail to identify true associations in rare-event settings.

The present study extends the *ttScreening* framework to binary outcomes with rare events. The original *ttScreening* method was developed for settings in which the outcome distribution is not extremely sparse. When the event rate is very low, the number of cases in each training or testing split can be small, leading to unstable association estimates and reduced ability to detect truly associated CpG sites. Rare outcomes also create challenges for conventional logistic regression, where maximum-likelihood estimates can be biased or may fail to exist under separation or quasi-separation. Logistic regression is the standard approach for examining associations between predictors and a binary outcome. However, in rare-event settings, fitted probabilities can be systematically underestimated, regression coefficients may exhibit substantial small-sample bias [11], standard errors can be inflated, and Wald-type tests may suffer from reduced power [12]. Firth’s bias-reduced logistic regression, originally proposed as a penalized likelihood method [13] and later shown to address separation by producing finite estimates [14], provides one way to reduce these problems. However, Firth-type estimators can also bias predicted probabilities toward 0.5 under strong outcome imbalance [15], and therefore their role in large-scale rare-event screening requires careful evaluation.

We propose two rare-event screening methods for high-dimensional EWAS: *Rare-Screening* and *Firth-ttScreening*. *Rare-Screening* is formulated as a differential methylation screening procedure. For each CpG site, DNA methylation is treated as a continuous response, and rare-event status is included as the group indicator. This formulation is natural for array-based EWAS and microarray-style analyses, where methylation values are commonly modeled as continuous outcomes and phenotype or group status is used to identify differential methylation. The method uses linear modeling with empirical Bayes variance moderation through the limma framework, which stabilizes CpG-specific variance estimates by borrowing information across sites [16]. Hellevik [17] argues that, despite ignoring the binary scale of the outcome, simple linear models can be very effective for screening when the main goal is to detect which variables are associated rather than to obtain precise probability estimates. Moreover, application of empirical Bayes moderation to the linear model [16] further enhances the power and stability of the estimates, making it well-suited for high-dimensional data with rare outcomes. *Firth-ttScreening*, in contrast, uses logistic regression with Firth’s penalty correction within a repeated training–testing framework. This method directly models the rare binary outcome while reducing small-sample and separation-related bias.

We evaluate the proposed methods through simulation studies under rare-event settings and compare their performance with benchmark procedures, including Bonferroni and FDR-based approaches. The primary objective is to assess whether the proposed procedures can retain truly associated predictors with improved sensitivity under severe event imbalance. We then apply the methods to an EWAS of DNA methylation at birth and early onset of menarche in the Isle of Wight birth cohort. The proposed procedures are intended to generate candidate sets of CpG for downstream validation and biological interpretation, especially in settings where the standard logistic regression of rare events with a single CpG is unstable or underpowered.

## 2 Methods

The *ttScreening* method offers a computationally efficient two-stage approach to variable screening [9]. The procedure begins by randomly splitting the dataset into two parts, with two-thirds for training and one-third for testing. Within each split, separate linear models, either ordinary least squares (OLS) or robust regression, are fitted for each predictor. Candidate predictors are then selected from the training dataset and further tested using the testing dataset. Rather than relying on a single pass through the data, the process repeats multiple iterations, and predictors that consistently show statistical significance across these iterations are selected based on a predefined frequency threshold.

We adopted the framework of the existing *ttScreening* method and developed two approaches, *Rare-Screening* and *Firth-ttScreening*, for binary outcomes with rare events.

### 2.1 Rare-Screening

*Rare-Screening* is formulated as a differential methylation screening procedure in which DNA methylation is treated as the response and rare-event status is treated as the primary predictor. Let *M_ij_* denote the methylation M-value for subject *i* at CpG site *j*, and let *Y_i_* ∈ 0, 1 denote the binary rare-event indicator. For each CpG site *j*, the marginal model is

(1) $$M_{ij}=\alpha_{0j}+\alpha_{1j}Y_{i}+\epsilon_{ij},\;\epsilon_{ij}∼N(0,\sigma_{j}^{2})$$

where $\alpha_{1j}$ represents the mean methylation difference between event and non-event groups at CpG site (j). When covariates are available, the model can be extended to an adjusted screening model:

(2) $$M_{ij}=\alpha_{0j}+\alpha_{1j}Y_{i}+\gamma_{j}^{⊤}Z_{i}+\epsilon_{ij},\;\epsilon_{ij}∼N(0,\sigma_{j}^{2})$$

where **Z***_i_* denotes measured covariates or adjustment variables, such as sex, batch effects, cell-type proportions, or surrogate variables. In the proposed screening procedure, CpG sites are evaluated using the coefficient corresponding to rare-event status, $\alpha_{1j}$.

We employed a linear modeling approach using the R limma package’s lmFit function, followed by empirical Bayes moderation with the eBayes function. Empirical Bayes moderation stabilizes the standard errors of the estimated coefficients by shrinking CpG-specific variances toward a pooled estimate across sites. This variance moderation is particularly useful in rare-event settings, where the number of event cases is small and site-specific variance estimates can be unstable.

Figure 1 summarizes the workflow of *Rare-Screening*. The method repeatedly resamples the original data with replacement to obtain robust screening statistics across iterations.

1. *Resampling:* Cases and controls are sampled with replacement at a 1:1 ratio.
2. *Linear modeling:* For each resampled dataset, we fit a linear model using the lmFit function in the R limma package to assess marginal associations between each CpG site and the outcome.
3. *Empirical Bayes moderation:* We then apply eBayes to moderate the standard errors by shrinking variances toward a pooled estimate for stable t-statistics and p-values.
4. *Within-iteration identification:* For each resample, a CpG site is declared significant if its p-value is less than 0.05.

This procedure is repeated for a prespecified number of iterations, such as 100. CpGs are chosen if they are significant for a specific number of times, referred to as the cutoff value, out of the total number of iterations.

### 2.2 Firth-ttScreening

This approach utilizes logistic regression with Firth penalty correction in a test-retest setting. The logistic regression model for a binary outcome can be written as

(3) $$logit\left\{P\left(y_{i}=1|x_{i}\right)\right\}=\beta_{0}+\beta_{1}x_{i},$$

where $\beta_{0}$ is the intercept and $\beta_{1}$ is the regression coefficient for the predictor. The predicted probability for the outcome for subject *i* is

(4) $$p_{i}=\frac{1}{1+exp\left(-\beta_{0}-\beta_{1}x_{i}\right)},$$

with log-likelihood

(5) $$l(\beta )=\sum_{i=1}^{n}\left[y_{i}log\left(p_{i}\right)+\left(1-y_{i}\right)log\left(1-p_{i}\right)\right],$$

where $\beta ={\left(\beta_{0},\beta_{1}\right)}^{⊤}$. Here, the predicted probability refers to the model-estimated likelihood that the outcome *y_i_* = 1 occurs for subject *i*, given their covariate values.

When the distribution of *y* is highly imbalanced and events are rare, the contributions of the event cases to l(β) are dominated by the many non-events. In such situations, the likelihood surface can become nearly monotone in certain directions of β, leading to strong small-sample bias and, in extreme cases, to quasi-separation or complete separation where the maximum likelihood estimate does not exist or is driven toward ±∞ [14].

Firth’s correction adds a penalty term to the log-likelihood to address issues such as small-sample bias and separation. The penalized log-likelihood is

(6) $$l_{F}(\beta )=l(\beta )+\frac{1}{2}log|I(\beta )|,$$

where I(β) is the Fisher information matrix and |I(β)| is its determinant. Logistic regression with Firth’s penalty can be conducted accordingly.

As shown in Figure 2, we first conducted logistic regression with Firth correction for all candidate predictors. A significance level of 0.05 was set for statistical significance based on the p-value. Then, the predictors that passed the significance test were carried to the next step. All samples were split into training and testing sets, with two-thirds of the sample as the training dataset and one-third as the testing dataset. For each predictor, it was recorded as “pass” only if it was statistically significant (*p* < 0.05) in both the training and test sets. This training–testing validation procedure was repeated for a prespecified number of iterations. Predictors were retained if they achieved “pass” in at least a prespecified number of iterations.

### 2.3 Simulation studies

To evaluate the performance of the proposed screening approaches, we conducted a series of simulation studies under high-dimensional settings. We first simulated the predictor matrix $X\in R^{n\times p}$, whose rows correspond to subjects and columns correspond to predictors. Each entry *X_ij_* is drawn independently from a standard normal distribution. The continuous outcome vector $Y\in R^{n}$ is then generated from the linear model

(7) $$Y_{i}=\sum_{j=1}^{p}\beta_{j}X_{ij}+\varepsilon_{i},\;i=1,\ldots ,n,$$

where the first five regression coefficients are set to *β_j_* = 10 for *j* = 1, …, 5 and the remaining coefficients are set to zero. The error terms *ε_i_* are assumed to be independent and identically distributed as 𝒩(0,1).

To create rare-event binary outcomes, we dichotomized the continuous outcome *Y* using cutoffs corresponding to the upper 5% and 1% quantiles of its empirical distribution, respectively. This yields binary variables indicating whether *Y_i_* falls in the top 5% or top 1% of the sample. The simulation studies were carried out under a range of scenarios with different sample sizes *n* and different numbers of predictors *p*.

In this simulation setup, the true signal set consists of the first five predictors, *T* = {1, …, 5}, and the remaining *p* − 5 predictors are null. For a given method and simulation replicate, let $\hat{T}$ denote the set of predictors declared significant by that method. We define

(8) $$\text{Sensitivity}=\frac{\left|\hat{T}\cap T\right|}{\left|T\right|},\;\text{Specificity}=\frac{\left|T^{c}\cap {\hat{T}}^{c}\right|}{\left|T^{c}\right|}.$$

Sensitivity is the proportion of true predictors selected, and specificity is the proportion of null predictors not selected.

For each combination of (*n*, *p*) and outcome prevalence, 5% or 1%, we generated data and applied all methods across 100 independent replicates. We then summarized the performance for each method and scenario by averaging sensitivity and specificity over the 100 replicates. The average sensitivity is

(9) $$\text{SEN}=\frac{1}{100}\sum_{i=1}^{100}{\text{Sensitivity}}_{i}.$$

Similarly, the average specificity is

(10) $$\text{SPE}=\frac{1}{100}\sum_{i=1}^{100}{\text{Specificity}}_{i}.$$

In *ttScreening*, the default selection frequency cutoff is 50% of resampling iterations, i.e., a predictor is retained if it is flagged as significant in at least half of the iterations [9]. To tailor this parameter for rare-event outcomes in *Rare-Screening* and *Firth-ttScreening*, we evaluated a grid of cutoff percentages *c* ∈ {10, 20, …, 90}, defined as the minimum proportion out of 100 resamples in which a predictor must be declared significant to be retained. We summarized sensitivity and specificity for each cutoff and identified the optimal value that provided the most favorable trade-off, maximizing true positive detection while minimizing false positives.

Finally, we compared the sensitivity and specificity of *Rare-Screening* and *Firth-ttScreening* at optimized cutoff values against two benchmark methods: Firth-corrected logistic regression with Bonferroni correction and FDR control.

## 3 Results

We summarized the sensitivity and specificity of the *Rare-Screening* method across cutoff thresholds, 50%–90%, for varying numbers of predictors (*p*) and sample sizes. For an event rate of 5% and *n* = 500, sensitivity declines modestly as the cutoff increases, whereas specificity rises slightly and then plateaus near 70–80%. The optimal cutoff is around 60% (Figure 3; Supplementary Fig. S1). When the sample size increases (*n* = 750, 1000, or higher), the optimal cutoff shifts to approximately 70–80% (Figure 4; Supplementary Fig. S2). Across all settings, both metrics are largely insensitive to increases in *p*, indicating strong stability and scalability in ultra-high-dimensional designs.Because a cutoff of 70% provided a consistently favorable balance across the evaluated sample sizes, we used this cutoff for *Rare-Screening* in subsequent comparisons.

For *Firth-ttScreening*, sensitivity decreased as the cutoff threshold increased, while specificity showed a modest rise before leveling off, mirroring the general pattern observed for *Rare-Screening*. The simulations suggest that, for smaller sample sizes such as *n* = 500, a cutoff of 30 provides a favorable balance between sensitivity and specificity, whereas for larger samples (*n* ≥ 750), a higher cutoff of 50, consistent with the default choice in the original *ttScreening* procedure, is preferable. These patterns are illustrated in Figures 5 and 6. Across all scenarios, the performance of *Firth-ttScreening* was largely unaffected by the number of predictors *p*, indicating good robustness in ultra-high-dimensional settings.

We compared *Rare-Screening* with cutoff 70 and *Firth-ttScreening* with cutoffs 30 and 50 with FDR and Bonferroni methods under different settings. Table 1 shows the sensitivity for the outcome with 1% event rate for all methods. For *n* = 1000 (10 events), *Firth-ttScreening* with 30 iterations yields the highest sensitivity (0.770–0.784) across *p*, followed by *Firth-ttScreening* with 50 iterations (0.670–0.680) and *Rare-Screening* at cutoff 70 (0.61–0.67). Multiple-testing procedures, FDR and Bonferroni, perform substantially worse in this sparse-outcome setting (0.050–0.202). When the sample size increases, the sensitivity increases in all methods. FDR and Bonferroni improve (0.650–0.830 and 0.774–0.910, respectively) but are still not as good as the screening-based methods. Overall, when the event rate is 1%, with such a small number of events, *Firth-ttScreening* with a cutoff of 30 is clearly the better method to screen for predictors. With increasing sample sizes and increasing number of predictors, *Rare-Screening* attains higher sensitivity compared to any other methods and attains 100% or close to 100% sensitivity. For all methods, specificity and accuracy are close to or above 99% regardless of the setting (Supplementary Tables 1, 2, and 3).

The sensitivity at a 5% event rate of our two screening methods, *Rare-Screening* with cutoff 70 and *Firth-ttScreening* with cutoffs 30 and 50, with FDR and Bonferroni adjustments under different scenarios is illustrated in Table 2. The results are similar to what was found for the 1% event rate. Both *Rare-Screening* and *Firth-ttScreening* have higher sensitivity compared to FDR and Bonferroni adjustments. As in the results for the 1% event rate, when the event rate is 5%, specificity and accuracy are close to 99% and above for all methods in any sample size and predictor number setting (Supplementary Tables 4, 5, and 6).

### 3.1 Case study on IOW female menarche data

Menarche, the onset of menstruation, is a key marker of female pubertal development and an indicator of reproductive health. Variation in age at menarche has important implications, as earlier onset before age 11 years has been consistently linked with earlier all-cause mortality, elevated risks of breast and ovarian cancers, diabetes, cardiovascular disease, and adverse psychosocial outcomes [18–20]. The association between genome-wide DNA methylation levels on CpG sites at birth and earlier onset of menarche is changing. This binary outcome, characterized as a rare event, presents significant challenges in efficiently screening CpG sites in epigenome-wide association studies.

The Isle of Wight (IOW) cohort is a population-based prospective birth cohort established in the United Kingdom among children born between January 1989 and February 1990, with repeated follow-up from infancy into adulthood [21]. The cohort provides a well-characterized longitudinal setting to examine the relationship between early-life epigenetic profiles and later developmental outcomes. For this study, age-at-menarche data were available for 319 participants. Early menarche was defined as menarche before 11 years of age and occurred in 13 participants (4.1%), reflecting the rarity of the outcome. Newborn DNA was extracted from dried blood spots on Guthrie cards and analyzed using Infinium EPIC BeadChip V1 (EPIC array) from Illumina (Illumina, San Diego, CA, USA). A total of 551,710 CpG sites remained after preprocessing and quality control [22]. Using these CpG sites as candidate predictors, we applied the *Rare-Screening* and *Firth-ttScreening* methods to identify DNA methylation markers at birth associated with early menarche onset.

Using the *Rare-Screening* method with a cutoff of 70, we identified 1031 CpG sites. By *Firth-ttScreening* with a cutoff of 50, we found 353 CpG sites, and with a cutoff of 30, we found 1144 CpG sites. We identified 316 common CpG sites as potential markers of early menarche between the CpG sites found using the *Rare-Screening* method with cutoff 70 and the *Firth-ttScreening* method with cutoff 50.

We then conducted a gene ontology analysis on the 316 common significant CpG sites that passed both the *Rare-Screening* and *Firth-ttScreening* methods to identify biological pathways associated with these CpG sites. We used the Gene Ontology Testing for Illumina Methylation Array Data (GOMETH) approach, implemented through the gometh function in the R package missMethyl [23]. This process maps significant CpG sites to Entrez Gene IDs, tests for GO term pathway enrichment, and gives the significantly associated biological pathways. Multiple biological processes were identified from the analysis based on their raw p-values, but were not statistically significant after FDR and Bonferroni correction. The top three biological processes were the extrinsic component of the omegasome membrane, the omegasome membrane, and the mitochondrial proton-transporting ATP synthase complex.

## 4 Discussion

This study proposed two novel screening frameworks, *Rare-Screening* and *Firth-ttScreening*, to address the challenges of variable screening in high-dimensional data with rare binary outcomes. Both methods extend the principles of the existing *ttScreening* framework by integrating statistical adjustments tailored for sparse event data. The *Rare-Screening* method leverages repeated resampling with replacement combined with linear modeling and empirical Bayes moderation to enhance signal detection stability, while *Firth-ttScreening* incorporates Firth’s bias-reduced logistic regression within the cross-validation approach to mitigate small-sample and separation bias. Through extensive simulation studies, we systematically evaluated their performance under varying event rates, sample sizes, and dimensionalities, and compared them with established multiple-testing correction procedures such as Bonferroni and FDR adjustments.

Importantly, the proposed procedures are intended as rare-event feature screening tools rather than final confirmatory inference procedures. Their primary goal is to retain a manageable set of candidate CpG sites with a high probability of including truly associated markers under severe outcome imbalance. Therefore, selected CpGs should be interpreted as candidates for downstream validation, prediction modeling, biological pathway analysis, or formal multiple-testing analysis, not as definitive discoveries with guaranteed FDR control.

Simulation results demonstrate that the *Rare-Screening* approach achieves higher sensitivity than FDR and Bonferroni corrections when event rates are low and sample sizes are modest. For example, at a 1% event rate, a minimum sample size of approximately *n* = 1000 (10 events) is required for stable performance (Table 1). As sample sizes and event rates increase, the sensitivity of *Rare-Screening* converges with that of FDR, Bonferroni, and *Firth-ttScreening*, indicating comparable performance under less sparse conditions. At a 5% event rate, *Rare-Screening* consistently outperforms FDR and Bonferroni in small to moderate samples (Table 2). With higher sample sizes and event rates, sensitivity becomes closer to or equal to 100% in *Rare-Screening* and *Firth-ttScreening*.

This study does not consider interactions between predictors, though such effects are likely to play a key role in complex traits. Interactions between environmental exposures and DNAm of CpGs and interactions among CpG sites may reveal important biological mechanisms beyond simple association studies. Future extensions of this work should incorporate interaction terms and adopt more sophisticated frameworks, such as multivariate screening or hierarchical modeling, to capture these relationships. Such developments would strengthen the biological interpretability of screening results while preserving the computational efficiency of the *Rare-Screening* framework.

To facilitate reproducibility and broader application, the proposed functions have been incorporated into the *ttScreening* R package, which is available through the Comprehensive R Archive Network (CRAN) [24]. The two methods also differ in computational efficiency. In a representative simulation with 10,000 predictors and 500 samples, *Firth-ttScreening* required 15.6557 minutes, whereas *Rare-Screening* required approximately 1.05 minutes. Compared with *Firth-ttScreening*, *Rare-Screening* offers a computational advantage in high-dimensional rare-event screening.

## 5 Conclusion

The proposed *Rare-Screening* and *Firth-ttScreening* methods provide practical screening strategies for high-dimensional studies with rare binary outcomes. Across simulation settings and an EWAS case study, the methods improved sensitivity compared with conventional FDR and Bonferroni approaches while maintaining high specificity. These findings support their use as efficient first-stage screening tools before downstream biological interpretation or multivariable modeling.

## Supplementary Material

Additional simulation results, including supplementary tables and figures, are provided in the Supplementary Information.

This is a list of supplementary files associated with this preprint. Click to download.

- rareeventpapersupp.pdf

## Figures and Tables

**Fig. 1:**
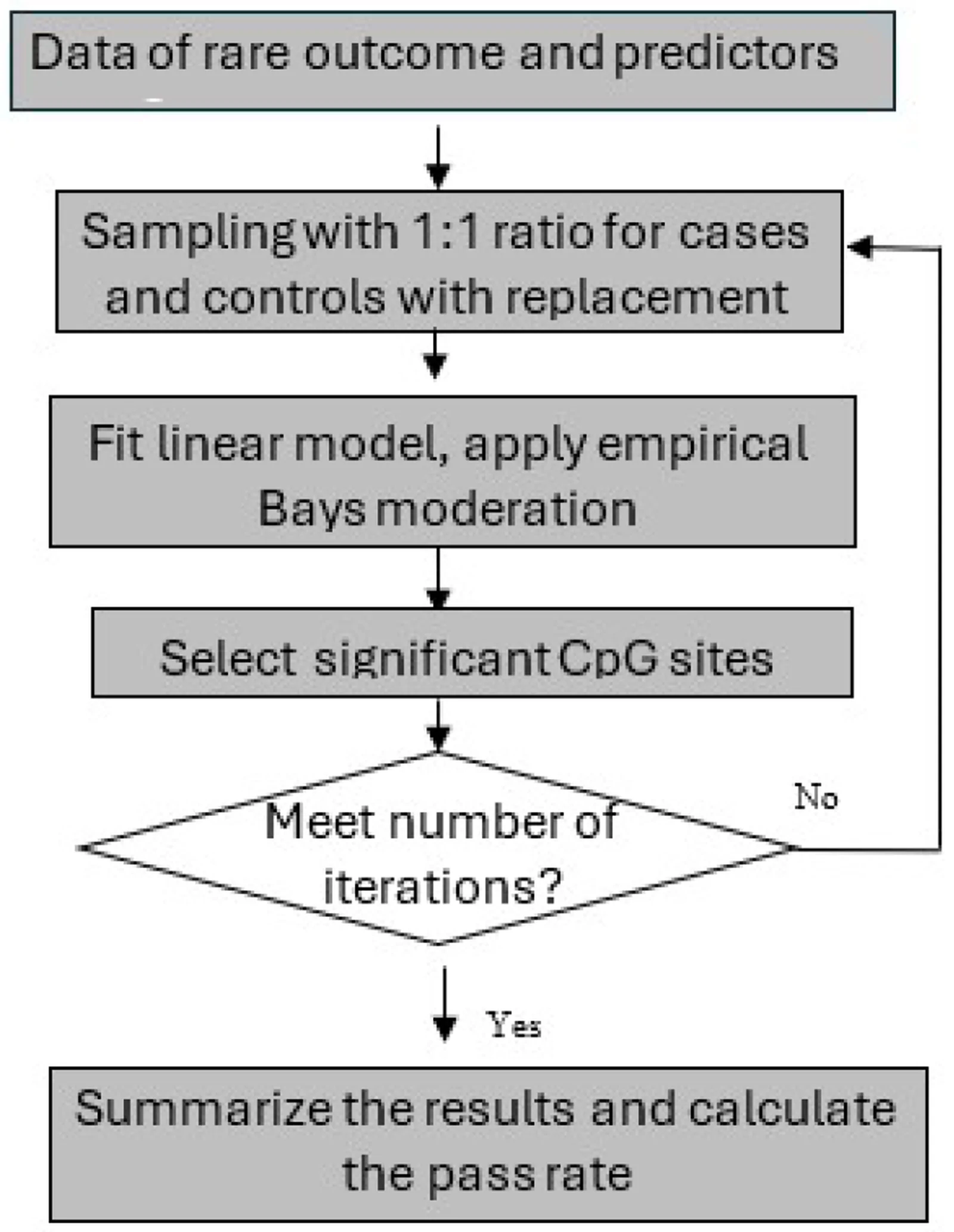
*Rare-Screening* method flowchart.

**Fig. 2:**
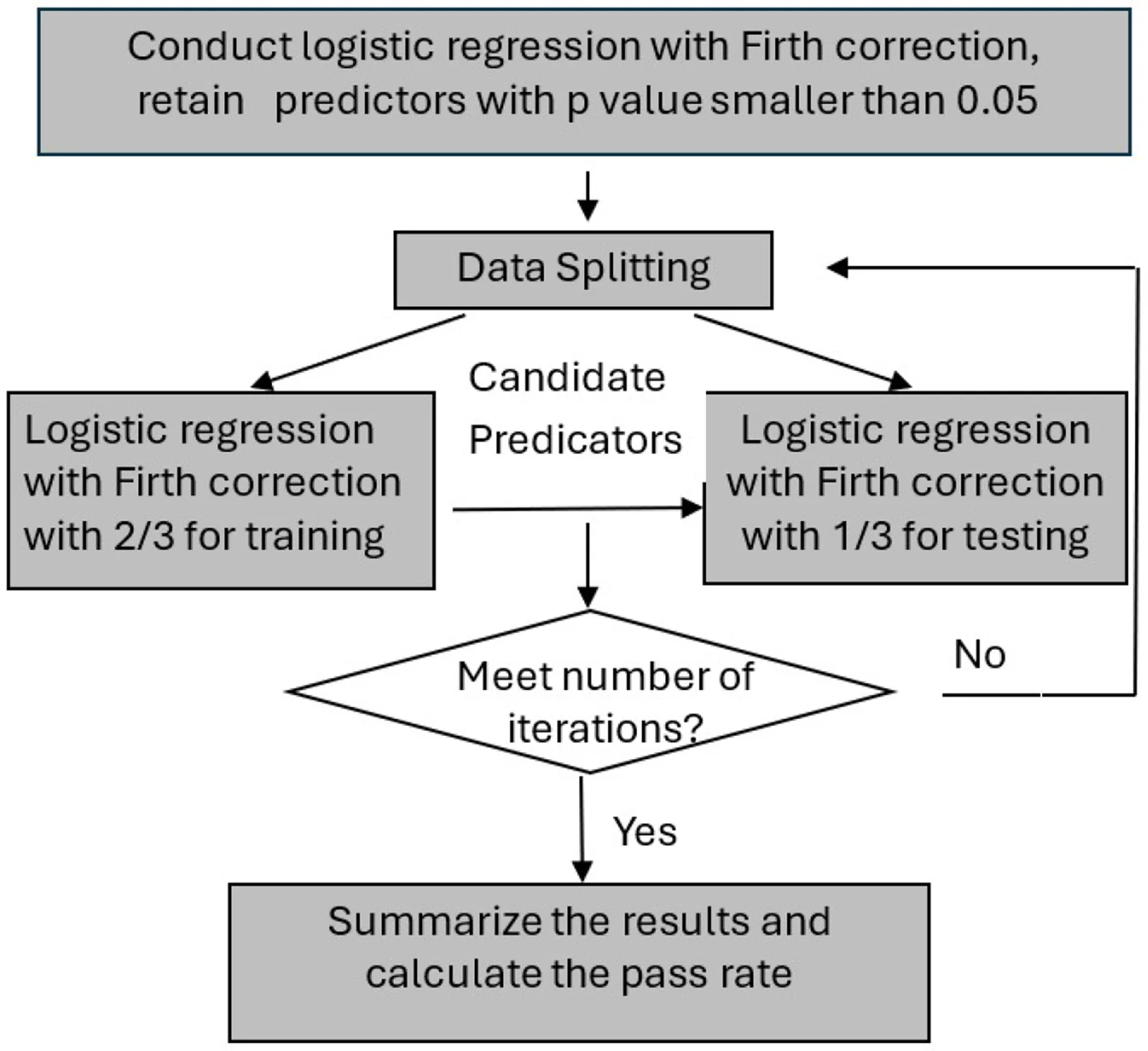
*Firth-ttScreening* method flowchart.

**Fig. 3:**
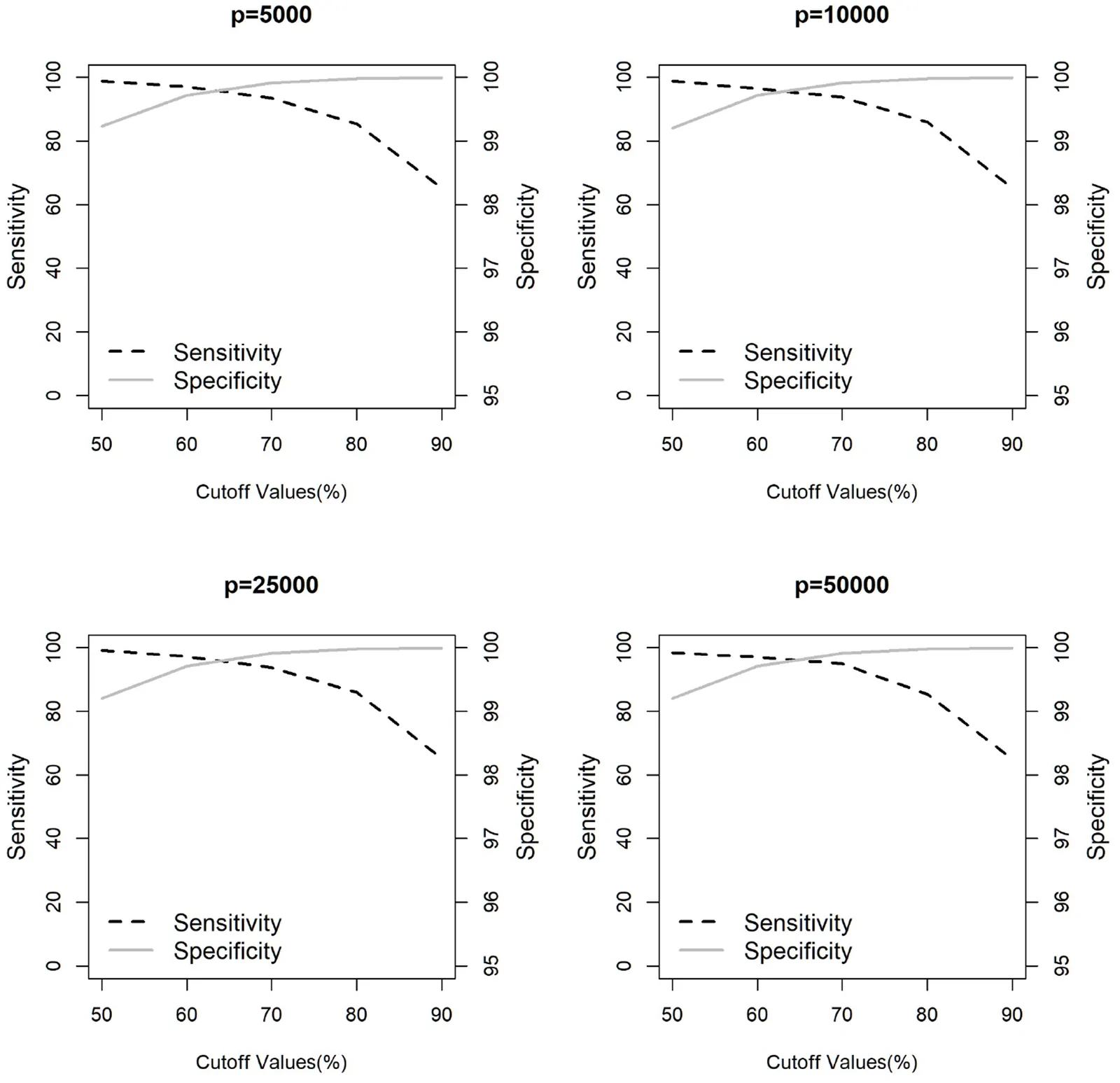
Sensitivity (dashed line) and specificity (solid line) of *Rare-Screening* at different cutoff thresholds when *n* = 500 for different numbers of predictors.

**Fig. 4:**
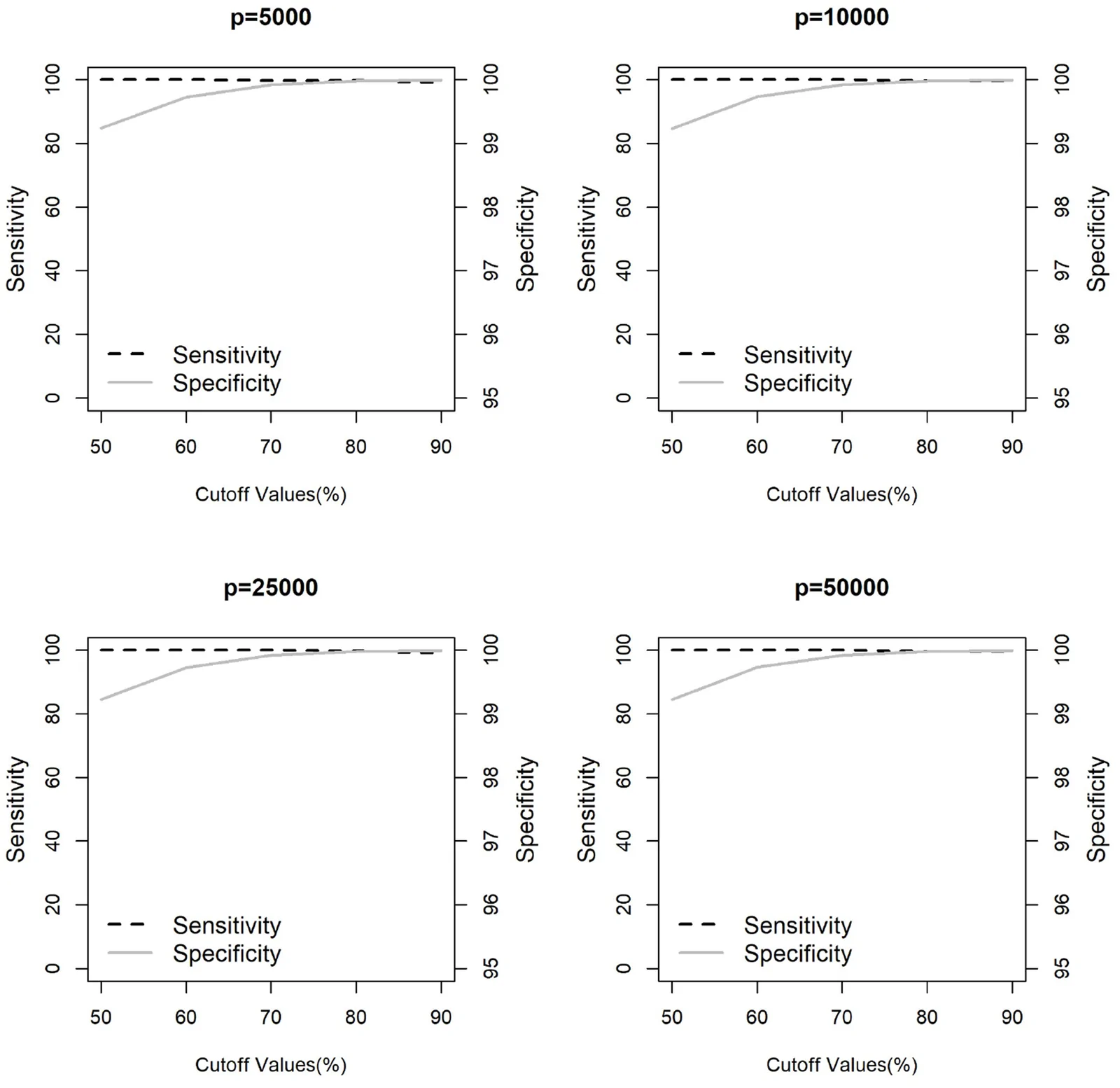
Sensitivity (dashed line) and specificity (solid line) of *Rare-Screening* at different cutoff thresholds when *n* = 1000 for different numbers of predictors.

**Fig. 5:**
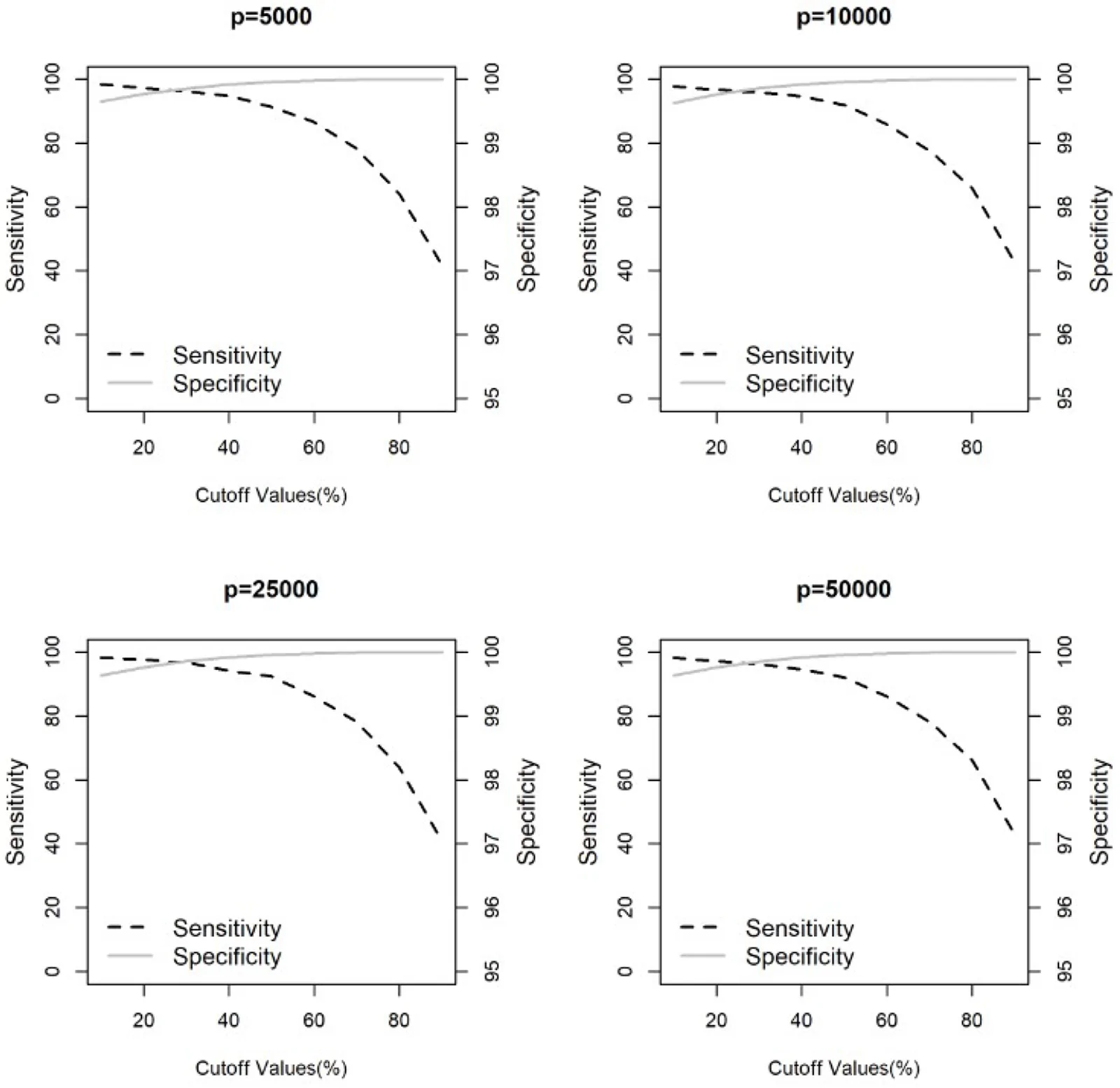
Sensitivity (dashed line) and specificity (solid line) of *Firth-ttScreening* at different cutoff thresholds when *n* = 500.

**Fig. 6:**
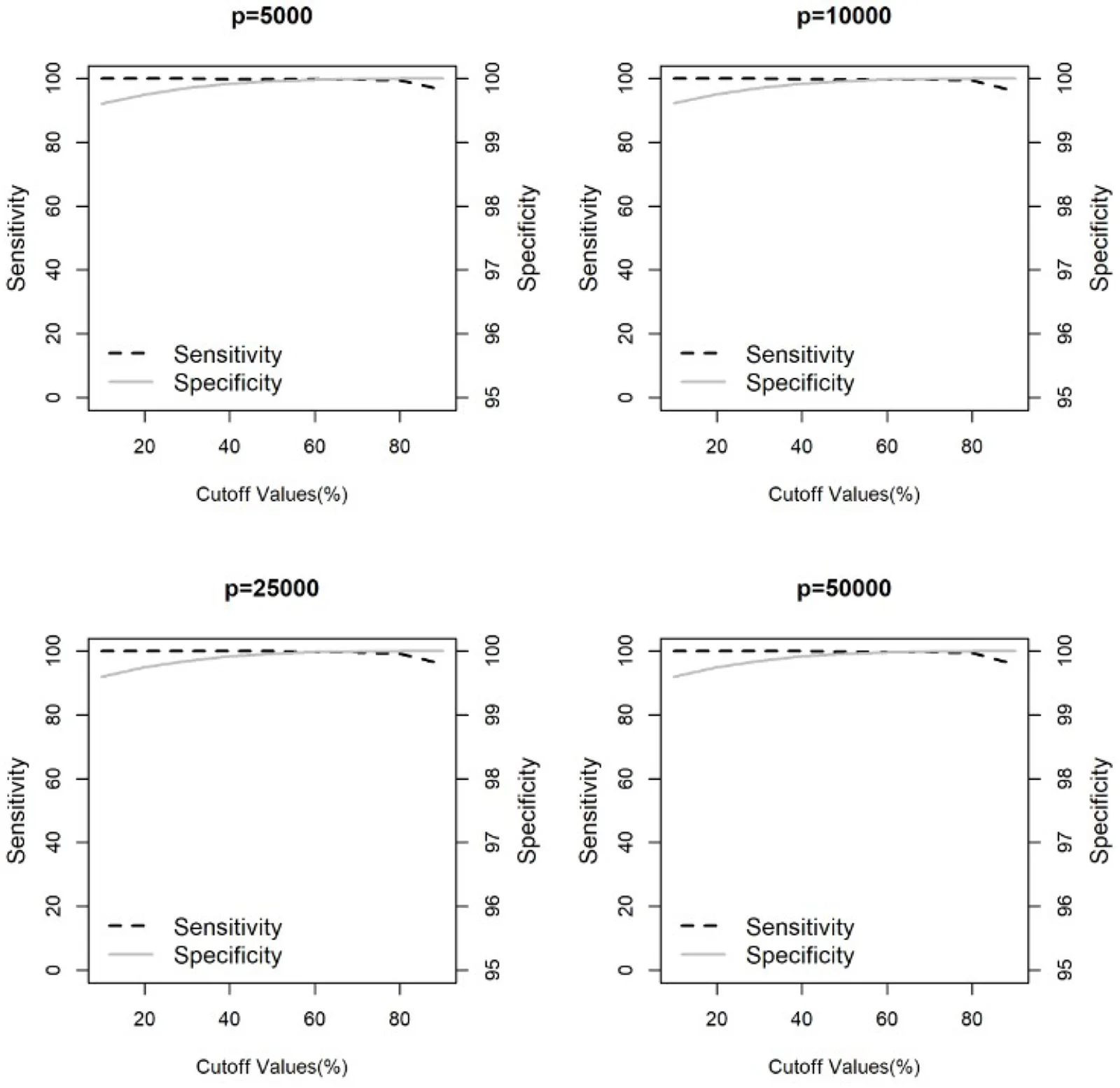
Sensitivity (dashed line) and specificity (solid line) of *Firth-ttScreening* at different cutoff thresholds when *n* = 1000.

**Table 1:** Comparison of sensitivity between *Rare-Screening, Firth-ttScreening* at different cutoffs, FDR, and Bonferroni methods at 1% event rate.

Predictor no.	Rare-Screening (70)	Firth-tt (50)	Firth-tt (30)	FDR	Bonferroni
**For sample size 1000 (10 events)**					
5000	0.65	0.67	0.78	0.202	0.164
10000	0.66	0.68	0.784	0.132	0.114
25000	0.61	0.672	0.77	0.092	0.076
50000	0.67	0.676	0.772	0.056	0.050

**For sample size 1500 (15 events)**					
5000	0.99	0.90	0.962	0.718	0.58
10000	0.99	0.89	0.96	0.642	0.50
25000	0.81	0.892	0.952	0.542	0.392
50000	0.99	0.884	0.96	0.458	0.336

**For sample size 2000 (20 events)**					
5000	1.00	0.91	0.986	0.83	0.91
10000	1.00	0.878	0.984	0.772	0.878
25000	1.00	0.828	0.988	0.702	0.828
50000	1.00	0.774	0.99	0.650	0.774

**Table 2:** Comparison of sensitivity at 5% event rate.

Predictor no.	Rare-Screening (70)	Firth-tt (50)	Firth-tt (30)	FDR	Bonferroni
**For sample size 400 (20 events)**					
5000	0.84	0.776	0.83	0.408	0.298
10000	0.84	0.780	0.83	0.292	0.206
25000	0.83	0.788	0.83	0.176	0.146
50000	0.83	0.784	0.84	0.094	0.082

**For sample size 500 (25 events)**					
5000	0.93	0.914	0.962	0.702	0.534
10000	1.00	0.920	0.958	0.618	0.454
25000	0.94	0.924	0.968	0.472	0.354
50000	1.00	0.922	0.962	0.390	0.298

**For sample size 750 (38 events)**					
5000	1.00	0.990	0.998	0.958	0.900
10000	1.00	0.990	0.996	0.932	0.858
25000	1.00	0.988	0.994	0.906	0.820
50000	1.00	0.986	0.996	0.870	0.782

**For sample size 1000 (50 events)**					
5000	1.00	0.996	1.000	0.996	0.996
10000	1.00	0.996	1.000	0.996	0.996
25000	1.00	0.996	1.000	0.996	0.982
50000	1.00	0.990	1.000	0.990	0.966

## Data Availability

The data analyzed in the case study are from the Isle of Wight birth cohort study (Arshad et al., International Journal of Epidemiology. 2018 A 1; 47(4):1043-4i). The data can be obtained from the cohort’s principal investigator, Dr. Hasan Arshad, upon request.Requests may be sent to s.h.arshad@southampton.ac.uk.

## Code availability.

The R package is available at https://cran.r-project.org/web/packages/ttScreening/index.html.
